# Stabilization of Copper-Based Biochips with Alumina for Biosensing Application

**DOI:** 10.3390/bios12121132

**Published:** 2022-12-06

**Authors:** Nour Beydoun, Yann Niberon, Laurent Arnaud, Julien Proust, Komla Nomenyo, Shuwen Zeng, Gilles Lerondel, Aurelien Bruyant

**Affiliations:** 1 Light, Nanomaterials & Nanotechnologies (L2n), CNRS-ERL 7004, Université de Technologie de Troyes, 10000 Troyes, France; 2 Phaselab Instrument SAS, 10325 Rosières-près-Troyes, France

**Keywords:** biosensors, surface plasmon resonance, plasmonic, biochips, copper

## Abstract

Surface plasmon resonance devices typically rely on the use of gold-coated surfaces, but the use of more abundant metals is desirable for the long-term development of plasmonic biochips. As a substitute for gold, thin copper films have been deposited on glass coverslips by thermal evaporation. As expected, these films immersed in a water solution initially exhibit an intense plasmonic resonance comparable to gold. However, without protection, an angle-resolved optical analysis shows a rapid degradation of the copper, characterized by a continuous angular shift of the plasmonic resonance curve. We show that copper films protected with a thin layer of aluminum oxide of a few nanometers can limit the oxidation rate for a sufficient time to perform some standard measurements. As the process is simple and compatible with the current biochip production technique, such an approach could pave the way for the production of alternative and more sustainable biochips.

## 1. Introduction

Due to its excellent chemical stability, biocompatibility, and optical properties, gold is a great material for the realization of plasmonic biosensors. The functionalization techniques for this material are well-established for a wide range of molecular and biological targets, and this efficient plasmonic metal is very resistant to oxidation. These characteristics explain its popular and almost exclusive use in consumable biochips employed for sensing in surface plasmon resonance (SPR) devices, although alternatives are now actively researched [1]. 

While surface plasmon resonance exhibits several advantages compared to other sensing techniques (real-time and label-free detection, kinetic and quantitative measurement with a high dynamic range, affinity measurement, high sensitivity), the use of gold, as in many other sensors, could pose a sustainability problem if the biochips were to be produced in large quantities. In addition to sensing application, the suitable properties of gold for controlled cancer treatment and drug delivery also make it a material globally superseding the other three major plasmonic materials; silver—which is also rare—and copper or aluminum. 

On the other hand, the two latter metals Al and Cu are much cheaper and more abundant than gold [2]. In this short list, aluminum is the second most abundant material of the upper continental earth crusts. It is gaining a large momentum of attention in the plasmonic community, as it can be relatively stable once its surface is sufficiently oxidized to form a protective layer of Al_2_O_3_. However, its optimal excitation is more centered in the long UV/blue range, where the sources, detectors, and materials are less accessible. Its plasmonic response cannot compete with gold in the red/NIR/IR regions where most of the studies and analyses are still performed. 

Copper, although less prevalent than aluminum, is still much more abundant in the earth’s crust than gold, and this material, sometimes referred to as an “urban mineral”, is rather efficiently recyclable [3]. A major and well-known obstacle to the use of copper in plasmonic applications stems from its poor chemical stability due to its rapid oxidation [4,5]. The oxidation of thin copper films has been notably investigated [6]; it is observed even in a normal atmosphere at room temperature, and it is known to be accelerated in water and buffer solutions commonly used in SPR detection. While the surface oxidation of Al prevents further oxidation of the metal, the copper oxide does not provide the same protection, and much research has been conducted to protect the copper layer. Recently, the possibility to use bare, ultra-flat, and defect-free copper crystal has been pointed out as a possible production solution [7] highlighting the role of defects and roughness in the oxidation process. However, the technique cannot be readily widely available in the standard process. Several authors also reported graphene as an anti-corrosive coating for the copper surface [8,9]. This approach is attractive since graphene can now be produced in relatively large quantities at a more affordable price. However, the direct coating of copper with a thin layer of protective materials remains desirable as it can be performed directly after the deposition of copper using the same process, in the same evaporation chamber. Atomic layer deposition of several thin oxide layers (ZrO_2_, HfO_2_, TiO_2_, ZnO, Al_2_O_3_) was notably used to successfully prevent copper oxidation with typical thickness layers of tens of nanometers [10]. The corrosion resistance attested by electrochemical measurement showed that Al_2_O_3_ provides one of the highest protections, in the initial stage of a few weeks, even in an alkaline solution. 

As the thickness of a protective dielectric spacer increases, so does the resonance angle, moving further away from the initial illumination angular condition of the SPR device. However, a benefit exists since the addition of a very thin (e.g., <10 nm) dielectric material of relatively high refractive index typically improves the sensitivity, as it increases an overlap integral for the plasmon field in the analyte region [11], although this sensitivity enhancement drops quickly above a limited thickness.

In our study, we explore the possibility of obtaining stable and highly resonant copper-based SPR chips, using the smallest protective layer possible of Al_2_O_3_ in order to maintain both a good interaction between the plasmonic resonance of copper and the analyte and a resonant angle compatible with conventional the SPR interrogation range. The protection was deposited just after the copper deposition in the same evaporation chamber. The resistance to oxidation was determined by a direct SPR measurement allowing a temporal follow-up of the oxidation that still appears for insufficiently protective layers. For a better estimate of the oxidation rate, a commercially available phase-sensitive SPR system was tested to enable an ellipsometric-like determination of the oxidation rate. While layers of about 2–3 nm of Al_2_O_3_ provided enough resistance to water for several hours, it was found that about 5 nm of Al_2_O_3_ was required to protect the copper over a long time in alkaline buffer solutions. It is worth noting, that the protection provided by the ultra-thin dielectric layer can be enhanced with oxygen plasma densification. This, however, requires an extra step in the procedure and is not considered necessary since 5 nm of oxide does not significantly alter the response of the copper chip.

## 2. Materials and Methods 

### 2.1. SPR Measurement Device

The SPR measurement was carried out using a compact phase-sensitive SPR from Phaselab Instrument SAS, adapted in our lab to accept a homemade coverslide covered with copper. The consumable gold-coated prism was replaced by a fixed BK7-prism and our thin copper plasmonic coverslip was coupled to this prism using a coupling oil. The slight optical mismatch created by this assembly replacing the gold-coated prisms created slight fringes on the obtained SPR curve without affecting the characterization of the chips. The illumination wavelength of the device was 670 nm and an automatized angle-scanning interrogation provided both amplitude and phase SPR curves. 

### 2.2. SPR Chip Design

The thin glass coverslide was covered with about 46 nm of copper followed or not by an ultrathin deposition of alumina. The estimation of the copper and Al_2_O_3_ thickness to be deposited was determined with a conventional multilayer code based on the transfer matrix method providing the complex reflectivity (amplitude and phase) of the designed biochips. The thickness providing the best-simulated coupling was found to be about 46 nm for the copper layer, although significant differences exist between the copper dispersions found in the literature for the optical indexes. By modeling, the sensitivity of the chip was determined to be about 110°/RIU, which was reasonably well confirmed by the experimental determination discussed later on.

### 2.3. Vapor Phase Deposition of Copper and Al_2_O_3_

The deposition of Copper and Alumina of high purity was carried out in the same chamber using a metal evaporator (Plassys MEB 400, France) with an evaporation rate of 0.1 nm/s. The thin film deposition occurred at a pressure of about 1.6 × 10^−5^ Torr. Cover slides from Agar scientific (L462264-15 # 1.5) were used as a substrate.

### 2.4. Microfluidic System Coupled to the Functionalized Biochip

The microfluidic systems were made using xurography of parafilm. It consisted of a cell of about 80 µm thick covered by a glass cover, equipped with an inlet and outlet, attached to the sample surface. A sufficient sealing was obtained by pressing the parafilm at 45 °C for ten minutes with the glass chamber cover.

### 2.5. Structural Characterization

**Figure 1:**
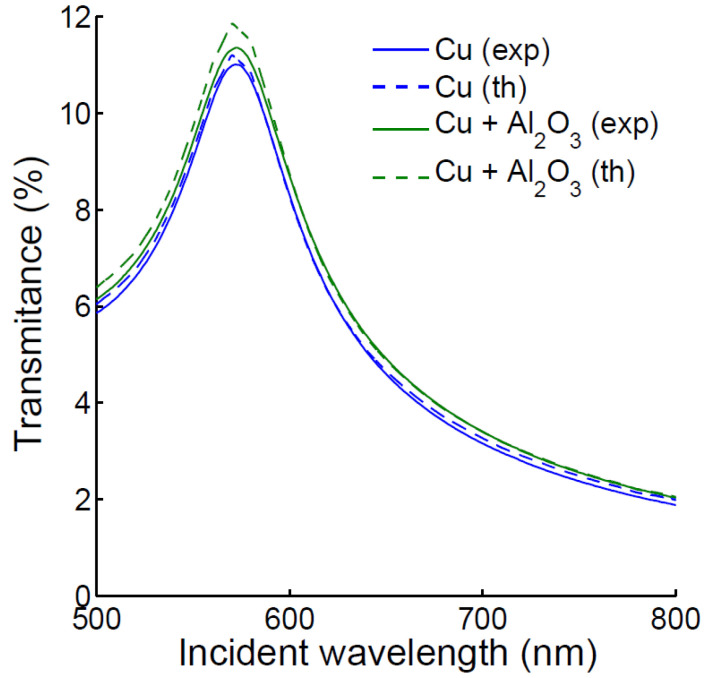
Experimental and simulated UV-visible spectra showing simulated and experimental transmittance of the optical chip coated with about 46 nm of Cu (blue) and with the addition of about 3 nm of Al_2_O_3_ Cu (green).

An estimation of the Cu chip thickness was carried out prior to the SPR experiments by comparing simulated transmission with ultraviolet (UV)-visible transmission spectrophotometry (Cary 100 UV-Visible Spectrophotometer from Analytic-Jena spectrophotometer). The transmission peak of Cu at 570 nm is found to be a good indicator of the copper thickness, and the addition of an ultrathin alumina layer slightly increases the magnitude of this peak by a fraction of a percent, both in simulation and experiment. The spectra are shown in Figure 1. A small increase is observed when a small Al_2_O_3_ layer of 3 nm is added on top of the glass coated with a 46nm thick copper layer.

The surface roughness of the films was investigated by both field emission scanning electron microscopy (HITACHI SU8030 SEMFEG) with an operation voltage of 0.5 V and AFM characterization (ICON Atomic Force Microscopes (AFM) from BRUKER) in “tapping” mode (intermittent contact).

## 3. Result and Discussion

### 3.1. Obtained Morphology

**Figure 2:**
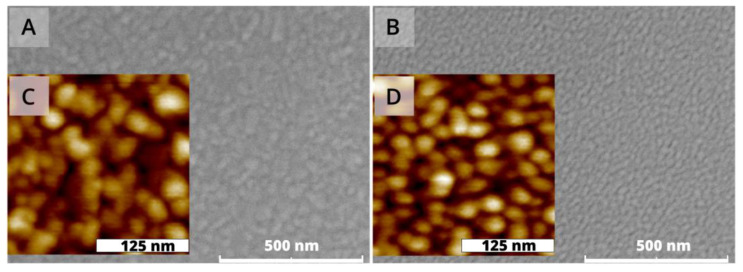
(**A**) Scanning electron micrograph of copper thin film deposited at room temperature. (**B**) Scanning electron micrograph of copper thin film deposited at room temperature protected by 3 nm of Al_2_O_3_. (**C**) Atomic force micrograph of copper thin film deposited at room temperature. (**D**) Atomic force micrograph of copper thin film deposited at room temperature protected by Al_2_O_3_. An RMS topography value of 1.0 nm was obtained on the bare copper.

Coverslides coated with 46 nm of copper were observed using SEM and AFM before and after alumina deposition. Figure 2A shows the SEM image of the bare copper thin films deposited on a glass substrate at room temperature. The film shows homogeneous surface morphology without cracks and pinholes. One can see from the SEM image that island-shaped nanoparticles are formed on the top surface. Meanwhile, the SEM images for copper protected by a 2 nm Al_2_O_3_ (Figure 2B) shows smaller grains on the surface, which can be attributed to the Al_2_O_3_ nano-islands formed on the copper surface. 

The results of the AFM characterization, whose images are also shown in Figure 2, give an estimation of the RMS roughness. The standard deviation on the bare copper topography is about 1.0 nm which is higher than that obtained through AFM analysis for the Al_2_O_3_, which is about 0.3 nm. This can be explained by the smoothing of the surface roughness upon the addition of Al_2_O_3_ [12].

### 3.2. SPR Measurement on Bare Copper Chips in Water 

**Figure 3:**
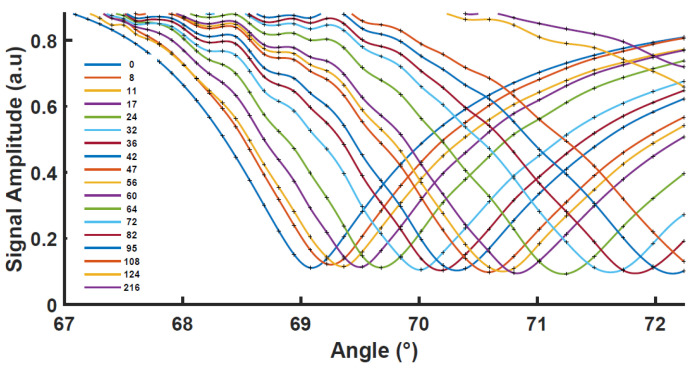
SPR curves for bare Cu thin films deposited on glass (46 nm) being in contact with deionized water. The time indicated in the legend is in minutes. The minimum amplitude is about 0.13 corresponding to a reflectivity of less than 2%.

The SPR curves in Figure 3 were recorded 24 h after the copper evaporation. During this time, the film was stored in a vacuum to prevent the diffusion of oxygen atoms in the air along the copper boundaries, which could increase the oxidation state of the copper film and decrease its quality. The bare copper-coated chips were immersed in water and SPR scanning curves were performed repeatedly from 5 min to 250 min after water immersion. As can be seen, the intensity of the resonant dip gradually shifts towards a high angle. 

The decrease of the SPR signal as a function of time indicated the plasmon coupling is reduced due to rapid film degradation. The resonant angle shifts from about 69.0 °C to 72.5 °C in 124 min; this SPR curve shift can be attributed to the oxidation taking place on the copper film surface. We can note that a similar shift for copper thin films in air was reported, but at a much slower rate by Chew et al. on single and polycrystalline Cu. The SPR measurement allowed the authors to estimate the optogeometrical parameters of the oxidation layer [13]. In the presence of water, this process is strongly accelerated here and, after one day, we can observe that the copper film is visible no more. The oxides formed on the copper surface are supposed to be comparatively stable in air in the absence of other influences, whereas upon water contact, the CO_2_ reacts to form carbonic acid (H_2_CO_3_) that is dissociated into hydrogen ions H^+^ and HCO_3_^−^. The great affinity of H^+^ to interact with the metal oxide forms water, leaving copper in dissociated ionic forms in the solution [14].

**Figure 4:**
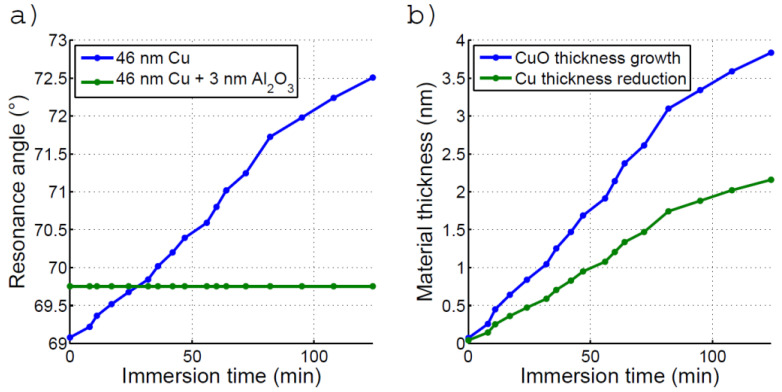
(**a**) Time evolution of the resonant angle for (**a**) the bare Cu thin film on glass and (**b**) the Cu thin film protected with 3nm of oxide, (**b**) Evolution of the CuO oxide growth at the bare Cu surface (in blue) and the corresponding reduction of the Cu metal (green) determined from the resonant angle change.

Assuming that, due to oxidation, a CuO film forms on top of the surface, and that the thickness of the film increases in time, the position of the resonant angle in time can serve to estimate the growth of this copper oxide layer and the corresponding decrease in the pure copper film. This angle position is shown in Figure 4a in blue, and the corresponding monitoring of the oxide layer thickness formed on the bare copper is shown in Figure 4b.

### 3.3. SPR Measurement on Protected Copper in Water

Adding an ultra-thin dielectric spacer tends to improve the sensitivity a bit. While thicker alumina protection can provide a longer standing protection to oxidation, it also induces a substantial angle shift in the SPR response towards a large angle, which may compromise the measurement and ultimately reduce the dynamic range and precision. Therefore, we tried to determine the minimal thickness of alumina ensuring sufficient protection for a test of at least 1 h. The films protected with 2 nm of Al_2_O_3_ only were found to lack chip-to-chip reproducibility in terms of oxidation resistance. However, with depositions of about 3 nm or more, a stable behavior was typically observed in water. The experimental position of the resonance angle as a function of time is plotted in Figure 4a (green) for this alumina thickness. The measurement was performed for about 2 h in ultra-pure water.

The good stability of the minimum angle position for the protected copper thin film indicates that an alumina layer of about 3 nm can protect the Cu thin films against oxidation in water for the time of an SPR measurement. As can also be seen, the alumina protection has shifted the resonance by about 0.68° at the beginning of the experiment, compared to the unprotected chips (68.9° to 69.9°). Given the chip sensitivity, this shift corresponds to a shift of about 6.2mRIU, which can be accounted for by simulation considering a thickness comprised between 2 and 3 nm (neglecting roughness).

### 3.4. SPR Measurement on Protected COPPER in PBS Solution

**Figure 5:**
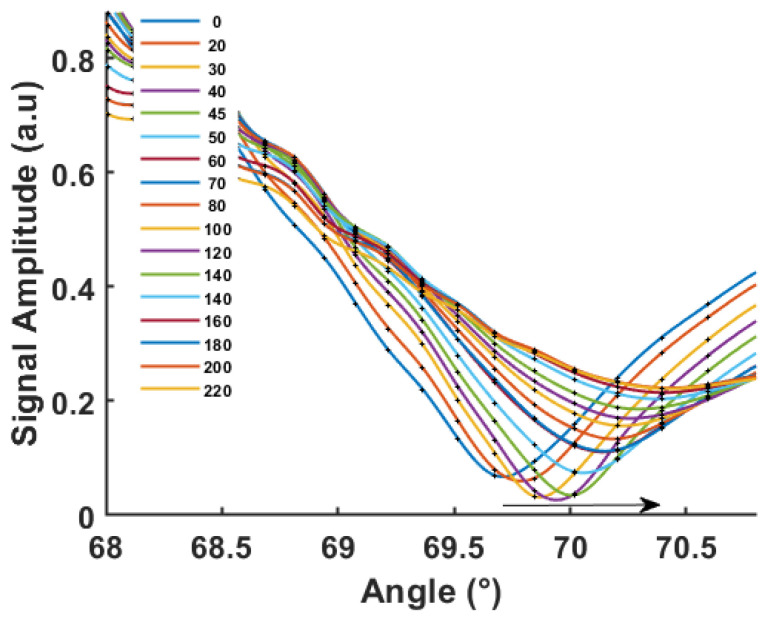
SPR curves in PBS solution for the Al_2_O_3_ protected copper for different time variations for the position of the minimum angle versus time for Cu thin films deposited on glass (46 nm) covered by 2 to 3 nm of Al_2_O_3_ being in contact with a PBS buffer electrolyte. The time is given in minutes in the legend.

To test the stability in real conditions, a standard buffer electrolyte solution of PBS was used at 20 mM concentration in replacement of water. The SPR curves obtained in this slightly alkaline solution over 3.5 h are shown in Figure 5. We observe an initial shift similar to the previous experiment, caused by the small alumina thickness. On the other hand, after an additional 200 min, the resonance dip becomes much less pronounced, as expected from a strong decrease of the Cu layer thickness or a strong degradation of the film quality. The shift is from about 0.03 RIU after 30 min to about 0.22 RIU within 220 min. The SPR resonance is still observable after a few additional hours but becomes wider and keeps shifting.

This SPR observation shows that protection of 2–3 nm of alumina is not sufficient to avoid Cu degradation in a conventional buffer solution. In addition, while the copper became greenish upon oxidation in the previous experiment, here the metal film is not more visible after sufficient exposition. The dissolution of Cu protected by Al_2_O_3_ in the PBS buffer analyte can be attributed to the initial dissolution of Al_2_O_3_. While oxygen is responsible for steel corrosion [15] and is also detrimental to copper plasmonic property, the case is different for Al_2_O_3_ oxide since the dissolved oxygen rather helps to strengthen the protective Al_2_O_3_ layer on aluminum. Therefore, the primary corrodents in the buffer electrolyte are the Na^+^ and Cl^−^ ions of the buffer that increase the conductivity of the solution and enhance the dissolution of alumina into complex ions Al(OH)_3_ and Al_2_(SO_4_)_3_. For aluminum, the corrosion mostly occurred by pitting, with a large number of pits that are formed and filled with a mixture of Al_2_O_3_, Al(OH)_3,_ and Al_2_(SO_4_)_3_. The resistance of different phases of alumina in salty water has been reported in several studies [16]. The defective nature of the oxide is usually responsible for the corrosion of the protected metal.

**Figure 6:**
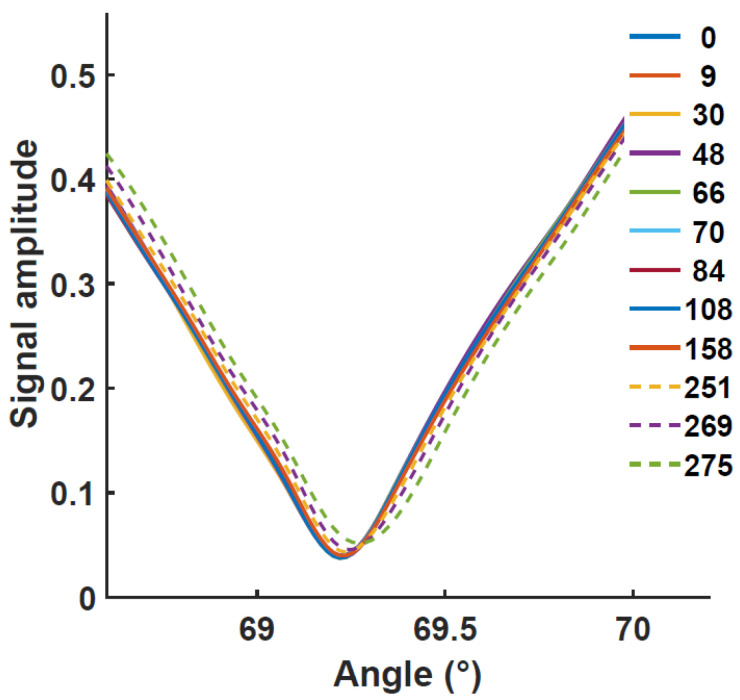
SPR curves for protected Cu thin films in contact with 20 mM PBS. The glass coverslide was coated with 46 nm of copper and protected with about 2 nm of Al_2_O_3._ The alumina surface was exposed to an oxygen plasma treatment of 12 min. After 250 min in the solution, an angle shift started to appear (dashed lines). The time is given in minutes in the legend.

To solve this issue, a test was performed on a layer of about 2.0 nm densified with an oxygen plasma. Oxygen plasma treatments increase the thickness of the aluminum oxide by increasing the formation of aluminum oxide on the surface. This post-deposition oxidation method further prevents the oxygen diffusion to the metal layer [17,18]. This test provided a stable resonant angle within the same PBS solution for about the same measurement time (~200 min). The curves are shown in Figure 6. No visible shift can be observed for the first three to four hours. However, a shift in the resonance angle is observed after about 250 min, after which time it accelerates.

**Figure 7:**
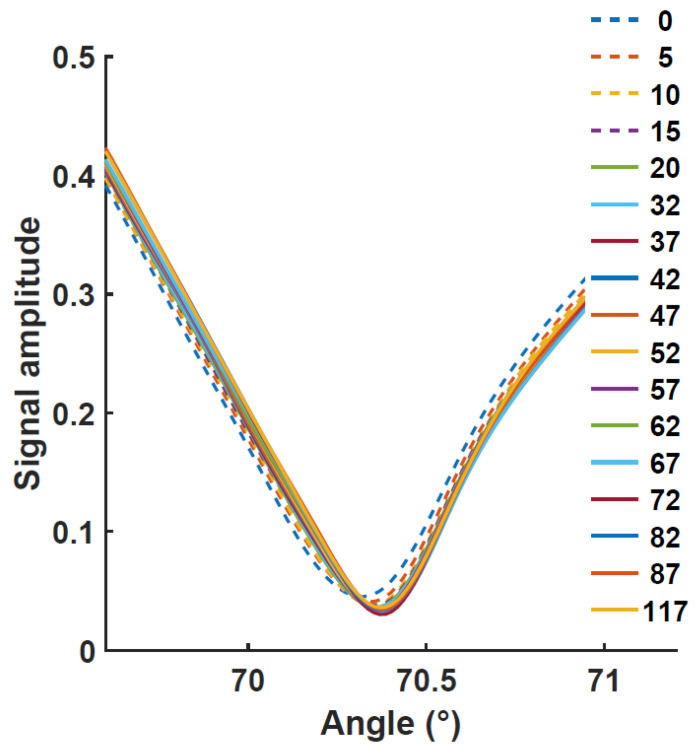
SPR curves for Cu thin films deposited on glass (46 nm) protected by 5 nm Al_2_O_3_ being in contact with 20 mM PBS. The time is given in minutes in the legend.

We can observe the relatively low value of the resonant angle near 69.2°, due to the smaller Al_2_O_3_ layer thickness. It should be noted, however, that a slight chip-to-chip variation in the resonance angle can be observed and that the roughness has an impact on the resonance position. While the plasma treatment makes the fabrication more complex since it requires an extra step and extra equipment, it does increase the protection and can avoid reproducibility issues. It is important to mention that we found that a slightly thinner oxide layer will not warrant stable behavior. Based on simulation, a thicker oxide layer of 5 nm can be deposited without detrimental effects and has a limited impact on the resonant angle shift. Figure 7 shows that the experimental angle shift induced by a 5 nm deposition still remains limited, with a resonant angle smaller than 70.5°. After a small shift was observed in the PBS solution, the resonant angle protection was found to have acceptable stability. The initial shift is relatively fast in the first 15 to 20 min (cf. dashed lines) after which the resonance angle becomes progressively more stable. Since no post-oxidation process was used for this chip, the initial shift observed in the PBS solution may be attributed to residual oxidation.

Given the results of this study, further functionalization tests (not reported here) were performed using 5 nm of Al_2_O_3_, with a post-oxidation process. The chips functionalized with APTES aminosilane did not show noticeable peak variation in the 120 min time scale.

## 4. Simulated Experiments 

The experimental studies showed a fast shift of the plasmonic peak for the chips coated with bare copper and immersed in water (Figure 3). To account for this effect, we consider that the Cu oxide progressively replaces the copper layer. Using the room temperature density and the molar volume of Cu and CuO, we determined that each nm of Cu was replaced by 0.563 nm of oxide. The possible presence of CuO_2_ was ignored as well as the effect of surface roughness.

**Figure 8:**
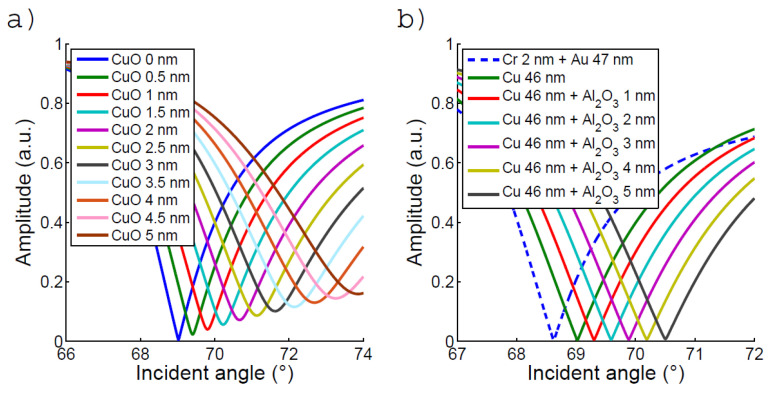
Simulation realized at 680 nm using the Fresnel coefficient for p-polarized light. (**a**) Simulation of an oxidized Cu 46 nm surface, for each 1 nm of CuO there is 0.563 nm of Cu removed. (**b**) Simulation of a 46 nm Cu surface passivated with different Al_2_O_3_ thickness. A simulation of a 47 nm Au surface with 2 nm of Cr adhesion layer is presented as a matter of comparison. The copper oxide growth in Figure 4b is interpolated from the position of the resonance given in this figure.

We calculated the signal of the reflectance of the SPR sensor working on the Fresnel coefficient for p-polarized light, for water as an upper medium. Figure 8 shows simulated reflectance measurements for the 46 nm Cu films. The redshift in the reflectance measurements corresponds to the copper oxidation versus time affecting the width of the plasmonic peak. The position of the resonant peak is relatively well accounted for by this simple calculation. It is worth mentioning that amplitude and phase information can be combined to rather precisely fit the data and determine effective complex permittivity values for the copper, as strong variations exist in the literature. This is, however, beyond the scope of this paper.

Table 1 provides the simulated sensitivity and figure-of-merit (FoM) of the chips of interest. The sensitivity is defined here as the variation of the resonance angle versus the refractive index shift occurring at the surface. The experimental determination of the sensitivity obtained for the Cu-based chip is reported in Appendix A. The FoM corresponds to the overall efficiency of a plasmonic interface [19]. It is the sensitivity divided by the full width at half the maximum of the associated absorbance curve (here 1 minus the scare value of Figure 8b). These optical simulations and results show that the theoretical sensing performance of Cu with the SPR is similar to that of Au at the considered wavelength (680 nm). The sensitivity can even be slightly better than gold if we consider that gold is coated on a chromium adhesion layer of 2 nm as commonly used. 

## 5. Conclusions

Copper-based SPR chips were designed and tested. The experimental optical sensitivity of 110°/RIU is comparable with that of gold. Without protection, a continuous shift of the resonance is observed in water, making it difficult to use for sensing application. An estimation of the oxide growth in time was proposed using a simple model based on Fresnel equations. Compared to water, the impact of a PBS buffer on the SPR resonance is stronger as not only a shift but a quick degradation of the resonance is observed in a few tenths of minutes. A stabilization of the resonance angle value is demonstrated both in water and in PBS, using an ultra-thin layer of alumina. In water, about 3 nm of alumina was sufficient to protect the surface for more than one and a half hours. Within a PBS solution, a plasma treatment has been used to further improve the resistance to oxidation while keeping the layer thin enough. A more direct approach consists of increasing the alumina thickness, keeping in mind that it will also substantially increase the resonance angle as well. However, 5 nm was found to be sufficient to substantially improve the stability of the surface in the PBS solution over a time of one hour or more while keeping the shift reasonably small. Without post-oxidation treatment, however, an initial shift can be observed. Therefore, better protection can be obtained by combining 5 nm thick alumina deposition with oxygen plasma treatment, without compromising sensitivity and by keeping a limited angle shift of about 2°. These results reinforce the idea that copper can effectively replace gold in SPR biochips without requiring advanced or complex processes. Further study at different pH levels and over longer periods of time could provide a better estimate of the achievable stabilization. 

## Figures and Tables

**Table 1:** A table presenting the resonance angle, sensitivity, and figure-of-merit for 47 nm of Au with 2 nm of Cr adhesion layer and 46 Cu surface passivated with different Al_2_O_3_ thickness.

Thickness (nm)	Cr:2 Au:47	Cu: 46	Cu: 46 Al_2_O_3:_ 1	Cu: 46 Al_2_O_3:_ 2	Cu: 46 Al_2_O_3:_ 3	Cu: 46 Al_2_O_3:_ 4	Cu: 46 Al_2_O_3:_ 5
**Resonance angle** (°)	68.62	69.02	69.30	69.59	69.89	70.19	70.50
**Sensitivity** (° RIU^−1^)	119.1	121.2	122.4	123.7	125.1	126.4	127.9
**FoM** (RIU^−1^)	25.6	27.32	26.81	26.33	25.86	25.42	25.01

## Data Availability

Data and Matlab software underlying the results presented in this paper are not publicly available at this time but may be obtained from the authors upon reasonable request.

## Appendix A. Sensitivity and Refractive Indices of the Layers

- Sensitivity measurement

The sensitivity of a 46 nm thick Cu surface passivated with 2 nm of Al_2_O_3_ on a glass prism was conducted using solutions of known refractive index. The results are presented in Table A1. Solutions of glycerol of 1, 5, and 10% in masse concentration in deionized water were used as this determination. The experimental sensitivity found is about 106° RIU^−1^ when the simulated counterpart is 108.5° RIU^−1^.

**Table A1:** Measures determining the chip sensitivity of a 46 nm thick Cu layer on glass, passivated with 2 nm Al_2_O_3_. The refractive index of the different solutions used was measured with a refractometer at 580 nm. We assume the Δn remains identical at the SPR wavelength. A linear fitting of the data gives a sensitivity of 105.9° RIU^−1^.

Glycerol Concentration (% m)	0	1	5	10
Refractive index (RIU-refractometer at 580 nm and 28 °C)	1.3321	1.3334	1.3384	1.3442
Resonance angle shift to reference (°)	0	0.1390	0.6670	1.2930

- Determination of the ratio 0.563

In Figure 8, the ratio 0.563 was calculated considering a Cu density of 8.96 g/cm^3^ and a CuO density of 6.315 g/cm^3^. The atomic mass of Cu was 63.546u and the molar mass for CuO was taken equal to 79.545 g/mol. The corresponding determined molar volume is 7.0922 cm^3^/mol and 12.5962 cm^3^/mol, for Cu and CuO, respectively, providing the determined ratio.

The reference of the refractive index used for the simulation is for Al_2_O_3_ [20], Au [21], Cr [22], Cu [22], CuO [23], BK7 glass [24], and water [25]. To account better for the observed dispersion in Figure 1 over the visible range, another complex index reference was used for the Cu [26].
